# Influence of synthetic and natural photosensitizers activated by photodynamic therapy on extrusion bond strength of fiber post to radicular dentin

**DOI:** 10.12669/pjms.37.7.4331

**Published:** 2021

**Authors:** Khalid H. Almadi, Mazen F. Alkahtany, Basil Almutairi

**Affiliations:** 1 Khalid H. Almadi, BDS, MS, FRCD(c) Department of Restorative Dental Science, Division of Endodontics, College of Dentistry, King Saud University, Riyadh, Saudi Arabia; 2 Mazen F. Alkahtany, BDS, MS, FRCD(c) Department of Restorative Dental Science, Division of Endodontics, College of Dentistry, King Saud University, Riyadh, Saudi Arabia; 3 Basil Almutairi, BDS, MSD, ABOD Department of Restorative Dental Science, Operative Division, College of Dentistry, King Saud University, Riyadh, Saudi Arabia

**Keywords:** Photodynamic therapy, Photosensitizers, Extrusion bond strength, Radicular dentin

## Abstract

**Objective:**

To assess the effect of different photosensitizers activated by low-level laser therapy on EBS of glass fiber post to radicular dentin.

**Methods:**

This study was conducted at King Saud University from January 2021 to March 202. Fifty maxillary central incisors were sanitized and decoronated. NiTi was used for mechanical instrumentation of the canal. All canals were shaped, cleaned and obturated with gutta-percha. Post space was made using peso reamers. Four Division of groups were made according to photosensitizers used (n=10). Group-1: MBP at 100mg/l, Group-2: Phycocyanin at 100mg/l, Group-3: CP at 500mg/l, and Group-4 toluidine blue photosensitizer (TB). Lasers were used for activation of photosensitizers. In Group-5 samples were irrigated using sodium hypochlorite NaOCl +17% EDTA. Posts were cemented and teeth sectioned into apical, coronal and middle. For EBS all samples were subjected to a universal testing machine. Fracture patterns were analyzed using stereomicroscope. To compare EBS at different segments One-way analysis of variance (ANOVA) and Tukey multiple comparison tests (*p*=0.05) was performed.

**Results:**

The maximum value of EBS was shown in Group-2 radicular canal treated with CP with 17% EDTA at all three levels cervical (8.61±1.32 MPa), middle (6.81±0.73 MPa), and apical (5.51±0.25 MPa). Similarly, the minimum value of EBS was displayed in Group-5 canal irrigated with 2.25% NaOCl +17% EDTA (control) coronal (6.10±1.77 MPa), middle (5.11±0.75MPa), and apical (3.60±0.94 MPa). Intragroup assessment disclosed a decrease in EBS from cervical one-third to apical one-third in all groups

**Conclusion:**

P, CP, and TB along with EDTA have the potential to be used as canal disinfectant and favors the bonding of GFP to radicular dentin using self-etch adhesive resin.

## INTRODUCTION

The success of root canal treatment is reliant on the reduction of microbial growth through irrigating solutions, proper instrumentation, and canal medicaments.^1^ Ideally sodium hypochlorite (NaOCl) at different concentrations is considered to be an irrigant of choice for canal disinfection along with ethylene diamine tetra-acetic acid (EDTA) to remove the smear layer after instrumentation. The smear layer is permeable to toxins, composed of organic and inorganic debris, and dwells bacteria. Current evidence advocates that it’s necessary to remove the smear layer before fiber post cementation to encourage close adaptation and penetration of cement in dentinal tubules.^2^ Moreover, available literature proclaims that though NaOCl as a canal irrigant minimizes bacterial load it has a detrimental effect on the internal wall of radicular dentin compromising the adhesion of fiber post.^3^–^6^

An alternative substitute for canal disinfection is photodynamic therapy (PDT) using different photosensitizers. Natural i.e., curcumin photosensitizer (CP) and chemically made photosensitizers i.e., methylene blue photosensitizer (MBP) has been already used in dentistry. MBP and CP are used in canal disinfection, on carries affected dentin (CAD), and as dentin and enamel conditioner and have shown promising outcome.^7^–^9^ However work by Sayhon et al., advocates that CP has an unfavorable effect on mechanical properties of dentin and it does not improve bond values of fiber post.^10^ Similarly, Strazzi Sayhon in his sequel study asserted that MBP for canal disinfection descends the bond value of fiber post to dentin.^11^

PDT works on the principle of absorption of a photon from a low-intensity visible light source resulting in reactive oxygen species (ROS) instigating lysis of bacterial cell wall through the process of oxidation.^8^ Ideally a photosensitizer should exhibit a low toxic effect on to host cell, produce ROS in a short period, water-soluble, and have an appropriate shelf life. None of the available photosensitizers confirm all the characteristics. But the available evidence suggests that toluidine blue (TB) and Phycocyanin (P) closely fall to these characteristics but their use in dentistry is scarce and not reported.^8^,^9^

To our knowledge from indexed literature, use of CP and MBP as canal disinfectant have shown to give conflicting outcomes. Moreover, the use of P and TB in sepsis of post space is still unprecedented. It is hypothesized that the use of conventional canal irrigant with EDTA will exhibit better extrusion bond values (EBS) at all three levels i.e., coronal, apical, middle apical compared to contemporary antimicrobial PDT+EDTA. Therefore, the present study aimed to assess the effect of different photosensitizers activated by low-level laser therapy on EBS of glass fiber post to radicular dentin.

## METHODS

Fifty maxillary central incisors were collected from clinical settings over 180 days. Teeth having a fracture, curved roots, or cracks were excluded. To maintain homogeneity, teeth having a canal length of 18mm and diameter of 2mm were placed in 0.5% thymol solution for disinfection at 4ºC for 72 hours. Decoronation of the teeth was done up to the cement-o-enamel junction buccolingually using a low-speed diamond saw (IsoMet 5000; Buehler). The study was conducted in King Saud University from January 2021 to March 2021under the approval of the Institutional review board number E-21-5829 and was reported following checklist for reporting invitro study (CRIS) guidelines.

Teeth were treated endodontically using K file#80 (Maillefer Instruments, Tulsa, USA). 1mm short of working length. Instrumentation of the canal was done mechanically via crown down technique using NiTi protaper system (Dentsply Maillefer). The shaping of the canal was done using S1, S2, and SX. The armamentarium of finishing files consisted of F1 and F2. During the cleaning and shaping process, the entire canal length was irrigated constantly using 1% sodium hypochlorite. Using paper points (Gapa Dent, Zhengzhou Smile Dental Equipment, Henan, PRC) the canal length was dried. Cone-shaped GP (Gapa Dent, India) along with sealers AH Plus (Dentsply, Konstanz, Germany) were used to fill the post space via lateral condensation. Peso reamer (Mani, ZZlinker, Shingai) # 4,3,2 was used to prepare post space.

Using K files #80 (Maillefer Instruments, Tulsa, OK, USA) teeth were treated endodontically 1mm short of working length. Using a protaper NiTi system (Dentsply Maillefer) via crown down technique mechanical instrumentation of the canal was done. The canal was shaped using S1, S2, SX shaping files and F1 and F2 finishing files following by constant irrigation with 1% sodium hypochlorite solution (8 ml). Paper points (Gapa Dent, Zhengzhou Smile Dental Equipment, Henan, PRC) were used to dry canals. Using a technique of lateral compaction canals were obturated with gutta-percha (Gapa Dent) and sealers AH Plus (Dentsply, Konstanz, Germany). All samples were kept in 100% humidification for seven days at 37ºC. Samples were placed vertically up to cement o enamel junction in polyvinyl pipes of diameter 4mm using cold cure acrylic resin. Post spacing was created using peso reamers (Mani, ZZlinker, Shingai) with numbers # 4,3, and 2. To handle the procedure professionally, recommended drills by the manufacturer of a number 100 fiber posts (Dentoclic glass fiber post) to a length of 10mm were used. All specimens were divided into five groups (n=10) based on the type of photosensitizer (PS) and conventional method on canal disinfection.

### Samples in Group-1 and Group-3

The canal was filled using methylene blue photosensitizer (MBP) 100mg/L (Sisco Research Lab. Pvt. Ltd, Maharashtra, India) prepared in 2% aqueous solution. A diode laser having a wavelength of 810nm was used to activate PS MBP. Irradiation time for the activation of oxygen species (O_2_) was 300 sec for MBP. Whereas, Curcumin PS in Group-3, was activated by 240 sec. of blue LED light (Clear Blue Digi 2.0) (λ 480 nm) irradiation. CP was used in a concentration of 50mg/L. A flexible fibre optic was used throughout the length of the canal for homogenous free radical formation. Seventeen percent EDTA was used to remove the smear layer throughout the canal length for 180 sec.

### Samples in Group-2 and Group-4

Phycocyanine (P) powder (Photoactive +, Weber medical, Germany) and Toluidine blue (TB) (Blue +T, Novateb, Iran) were prepared freshly in an aqueous solution of concentration 100mg/L each. TB and P were poured in the canal length and were activated using a 635-nm diode laser (Konftec, Taiwan) with an output of 220 mW in continuous mode for 180 sec. The power density of the device was 0.33 w/cm2. Laser power was checked with a power meter (Coherent, USA) before experiments. 17% EDTA was used to remove the smear layer throughout the canal length for 180 sec. (Table-I).

**Table I:** Details regarding photosensitizers used in the present study.

*Class*	*Example*	*Charge*	*Excitation Maximum (nm)*	*The concentration of PS mg/L*
Phenothiazinium	Methylene blue	Cationic	632	100
	Toluidine blue	Cationic	410	100
Natural Photosensitizers (PS)	Curcumin	Neutral	547	500
	Phycocyanin	Neutral	670	100

### Samples in Group-5

The specimens in Group-5 were irrigated using 2.25% sodium hypochlorite (NaOCl) (Vishal DentoCare Private, Ltd, India) and 17% EDTA (Pulpdent Corporation, Watertown using a disposable needle syringe of 25 gauge in a to and fro motion 1mm short of working length.

After canal disinfection in all groups. qll specimens were washed using distilled water and dried using paper points.70 % ethanol was used to clean the glass fiber post (GFP) (Swastik Dentomed Device, India) and fitted in the canal. The canal space was filled with self-etch resin cement Rely X Unicem (3M ESPE, St. Paul, MN, USA) and light-cured (LED B Woodpecker Light Cure Suz - Dent (India) Private Limited) using blue halogen light for 30 secone. For 48 hours all specimens were placed in the humid environment before performing EBS testing

Using a diamond bur all samples were sectioned (1 mm segment each) into coronal, middle, and apical. A total of 180 segments were prepared from 50 samples. For EBS all samples were subjected to a universal testing machine (1 mm/min) (Zwick/Roell Z050, Germany. Force was applied in a coronal apical direction. The force required to debond the fiber post from radicular dentin was measured in megapascal (MPa).


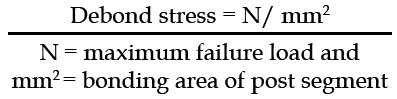


Using stereomicroscope at 50x magnification fracture patterns of debonded surfaces were analyzed. The patterns of fracture were categorized into cohesive, adhesive, and admixed. One-way analysis of variance (ANOVA) and Tukey multiple comparison tests (*p*=0.05) was done to compare EBS at different segments of the root structure.

## RESULTS

The highest extrusion bond strength (EBS) was shown in Group-2 radicular canal treated with CP with 17% EDTA at all three levels cervical (8.61±1.32 MPa), middle (6.81±0.73 MPa), and apical (5.51±0.25 MPa). Similarly, the lowest EBS was displayed in Group-5 canal irrigated with 2.25% NaOCl +17% EDTA (control) coronal (6.10±1.77 MPa), middle (5.11±0.75MPa), and apical (3.60±0.94 MPa). Homogenous distribution of data was assessed using Leven’s test. EBS values of all groups were presented in Table-II.

**Table II:** Means and Standard deviations (SD) of extrusion bond strength (MPa) values among experimental groups at cervical, middle, and apical levels of root.

*Groups*	*Cervical*	*Middle*	*Apical*
***Group-1:*** Methylene blue photosensitizer (MBP) + 17% EDTA	6.54±0.21 ^b, A^	5.59±0.41 ^b, A^	3.62±0.78 ^b, B^
***Group-2:*** Phycocyanin (P)+ 17% EDTA	8.21±0.35 ^a, A^	6.21±1.28^ a, A^	5.13±0.22^ a, B^
***Group-3:*** Curcumin photosensitizer (CP) (CP)+ 17% EDTA	8.61±1.32 ^a, A^	6.81±0.73 ^a, A^	5.51±0.25 ^a, B^
***Group 4:*** Toluidine Blue photosensitizer (TB)+ 17% EDTA	8.51±0.74 ^a, A^	6.95±0.91 ^a, A^	5.18±0.74 ^a, B^
***Group-5:*** 2.25% NaOCl +17% EDTA (control)	6.10±1.77 ^b, A^	5.11±0.75 ^b, A^	3.60±0.94 ^b, B^

Different superscript lower-case alphabets denote statistically significant difference within same column (p<0.05), Data with different upper-case alphabets denotes significant difference within each row. (p<0.05).

**Table III:** Fracture Pattern according to failure type.

*Groups*	*Root segment*	*Cement / DentinAdhesive*	*Cement / PostCohesive*	*Admixed*
Group-1	Coronal	60%	10%	30%
	Middle	70%	10%	20%
	Apical	70%	20%	10%
Group-2	Coronal	30%	60%	10%
	Middle	80%	10%	10%
	Apical	70%	20%	10%
Group-3	Coronal	30%	50%	20%
	Middle	50%	30%	30%
	Apical	70%	10%	20%
Group-4	Coronal	10%	20%	70%
	Middle	50%	20%	30%
	Apical	90%	10%	10%
Group-5	Coronal	70%	-	30%
	Middle	50%	20%	30%
	Apical	80%	10%	10%

Intragroup assessment disclosed a decrease in EBS from cervical one-third to apical one-third in all experimental groups. At the apical region, a statistically significant difference was displayed in comparison to middle and cervical root sections among all groups (p<0.05).

Intergroup comparison unveiled comparable extrusion bond strength in control group canal disinfected with 2.25% NaOCl with 17% EDTA and Group-1 post space disinfected using MBP at all three levels of root structure coronal, middle, and apical. Similarly, samples in Group 2, 3, and 4 canal space disinfected with Phycocyanin (P) with 17% EDTA, CP with 17% EDTA and Toluidine Blue photosensitizer (TB) + 17% EDTA displayed statistically significant difference to control and specimens in Group-1 at all three levels (p<0.05).

Forty-five failures were observed between dentin and adhesive interface. Failure between adhesive and cement interface was dominant among all interventional groups. Adhesive failure was followed by post cement failure type i.e., cohesive.

## DISCUSSION

The current study was assumed on the hypothesis that the use of conventional canal irrigant with EDTA will exhibit better extrusion bond values (EBS) at all three levels i.e., coronal, apical, middle compared to contemporary antimicrobial PDT+EDTA. To our surprise, a partial acceptance of the supposition was noted as antimicrobial PDT using CP, P and TB displayed better EBS to conventional 2.25% NaOCl +17% EDTA and MBP at all three levels of canal length. The bond power between GFP and radicular dentin was assessed using EBS. The test is dependable as it replicates oral condition and transmits stress equally along the long axis of radicular dentin.^12^,^13^ The test has a low failure rate, is highly sensitive and measures three different portions from a single root structure. It also provides a comparative analysis with other investigational groups.^14^

In the present study, two different classes of photosensitizers were assessed. MBP and TB from class Phenothiazinium whereas, CP and P from natural class photosensitizers. Both natural photosensitizers (CP [Coronal 8.61±1.32 MPa; middle 6.81±0.73 MPa; apical 5.51±0.25 MPa]) and (P [Coronal 8.21±0.35 MPa; middle 6.21±1.28 MPa; apical 5.13±0.22 MPa]) showed comparable EBS at all levels of radicular dentin. The production of singlet oxygen varies for every different photosensitizer.^15^ The quantity of singlet oxygen is measured by quantum yields. Literature reports that P when activated has the lowest oxygen production with quantum yields hence it does not interfere with the polymerization of resin to radicular dentin favoring increase bond values.^16^ Moreover, CP having an anionic nature binds with Calcium (Ca++) in radicular dentin encouraging retention of fibre post to dentin structure.^10^ Since, both natural photosensitizers (CP and P) are hydrophobic and polyphenolic these characteristics might have attributed to improving EBS.^7^ These outcomes should be implemented in the clinical setting with caution as still use of P is unprecedented in dentistry. Apart from natural photosensitizers, TB (coronal 8.51±0.74 MPa, middle 6.95±0.91MPa, apical 5.18±0.74MPa) an artificial photosensitizer displayed bond values comparable to CP and P. A logical explanation for this outcome is TB at this concentration (100mg/l) when activated at 638nm wavelength, might have boosted cross-linking of collagen fibrils in the dentin to resin providing a better outcome.^17^,^18^ Moreover, diffusion capacity of TB, hydrophobic nature and light energy fluency of TB may have resulted in favorable EBS.

Among all the photosensitizers MBP had the lowest EBS at all three parts of the root segment (coronal 6.54±0.21MPa; middle 5.59±0.41 MPa and apical 3.62±0.78 MPa). MBP is cationic. Its affinity with anionic molecules in dentin i.e., calcium and phosphate might cause precipitation on the dentinal walls deteriorating EBS.^19^,^20^ Furthermore, MB is hydrophilic. The hydrophilicity of MB at a higher concentration may influence the absorption of water from inter and intratubular part of dentin compromising EBS.^20^,^21^ These explanations have already been discussed by Sayhon et al. 19

A descend in EBS values was observed among all investigated groups. Poor penetration of photosensitizers, variations in the anatomy of root structure, small dense and less dentinal tubules at the apical region may have contributed to this outcome.^22^,^23^ Cement dentin interface failure was observed in the majority of all the samples, suggesting interface to be the weakest between post and radicular dentin.^22^,^23^

### Limitations of the study

Within the limitations of the study, atomic force microscopy of radicular dentin structure pre- and post-photosensitizers usage needs assessment. Moreover, topographic analysis of dentin and its effect after using different photosensitizers along with their effect on the micromechanical structure requires further valuation. De bonded surfaces should be assessed using dispersive spectroscopy. More clinical and lab-based studies need to be performed to extrapolate the findings of the present study.

## CONCLUSION

P, CP, and TB along with EDTA have the potential to be used as canal disinfectant and favors the bonding of GFP to radicular dentin using self-etch adhesive resin. Use of MBP and conventional method of canal disinfection using 2.25% NaOCl +17% EDTA should be used with cautions in dental clinical settings.

### Authors’ Contribution:

**KHA, BA MFA:** Data collection, study design, manuscript writing, final manuscript approval.

**KHA, BA:** Data collection, manuscript approval, and data interpretation.

**KHA, MFA:** Data collection, writing, revision of the manuscript, editing, and final manuscript approval.

All authors are responsible and accountable for the accuracy and integrity of the work
